# Supplementary material for: Аmygdala DEGs are associated with the immune system function: A comparative transcriptomic study of high- and low-excitability rat strains
Source: PLoS One. 2025 May 15;20(5):e0323325. doi: 10.1371/journal.pone.0323325 (PMC12080833; doi:10.1371/journal.pone.0323325)
Supplement: Attachment — Submitted filename: plos one.docx [file pone.0323325.s003.docx]

Abstract need more information with figures

Number of animals used is low and is the power analysis used before starting the experiment

Methodology lacks reference citation

Table 1 and table 2 looks like a general information

- Without employing functional assays (such as qPCR, protein quantification, or immunological assays) to validate important differentially expressed genes (DEGs), the study only used transcriptome data (such as RNA-Seq).


  • Despite the rat genome's comparatively good annotation, some genes or isoforms—especially those pertaining to immune-neural interactions—may still be missing functional annotations. This may restrict how DEGs are interpreted and how they might affect immune system function.
- The study might find DEGs linked to immune function, but it doesn't investigate the fundamental processes that connect immune system control with amygdala excitability. The results might not give a good picture of how these DEGs affect immune-neural connections in the absence of mechanistic knowledge.
