# Supplementary material for: Аmygdala DEGs are associated with the immune system function: A comparative transcriptomic study of high- and low-excitability rat strains
Source: PLoS One. 2025 May 15;20(5):e0323325. doi: 10.1371/journal.pone.0323325 (PMC12080833; doi:10.1371/journal.pone.0323325)
Supplement: Attachment — Submitted filename: Response to Reviewer2.docx [file pone.0323325.s004.docx]

Reviewer #2: Abstract need more information with figures

Number of animals used is low and is the power analysis used before starting the experiment

We have updated the abstract to include information regarding the sample size.

We did not perform a power analysis due to the high cost of RNA-seq, which limited the number of animals we could use for the experiment.

We understand your concern regarding the relatively small sample size (n=5 per group). However, RNA-seq is a very expensive method, and such limitations prevent us from using a larger number of animals for analysis. While more samples are always preferable for ensuring more reliable results, statistical methods do allow the use of n=5. Additionally, it is worth noting that RNA-seq studies often use even fewer biological replicates in global gene expression experiments. According to the "Best Practices for RNA Sequencing" from the ENCODE Consortium (<https://www.encodeproject.org/documents/cede0cbe-d324-4ce7-ace4-f0c3eddf5972/@@download/attachment/ENCODE%20Best%20Practices%20for%20RNA_v2.pdf>), experiments can be conducted with as few as two biological replicates.

Moreover, RNA-seq studies published by other research groups commonly use sample sizes of n=5-6 or even n=3, as seen in the following examples:

- Zhao S, Fung-Leung WP, Bittner A, Ngo K, Liu X. Comparison of RNA-Seq and microarray in transcriptome profiling of activated T cells. PLoS One. 2014 Jan 16;9(1):e78644. doi: 10.1371/journal.pone.0078644. eCollection 2014. ("There were a total of six time points, with two biological replicates per time point.")
- Dias C, Feng J, Sun H, Shao NY, Mazei-Robison MS, Damez-Werno D, Scobie K, Bagot R, LaBonté B, Ribeiro E, Liu X, Kennedy P, Vialou V, Ferguson D, Peña C, Calipari ES, Koo JW, Mouzon E, Ghose S, Tamminga C, Neve R, Shen L, Nestler EJ. β-catenin mediates behavioral resilience through Dicer1/microRNA regulation. Nature. 2014 December 4;516(7529):51–55. doi:10.1038/nature13976. (n=3 was used).
- Nahvi, Roxanna J., et al. "Transcriptome profiles associated with resilience and susceptibility to single prolonged stress in the locus coeruleus and nucleus accumbens in male sprague-dawley rats." Behavioural brain research 439 (2023): 114162. (n=5)

We hope this explanation clarifies our rationale for the chosen sample size and demonstrates that it is in line with current practices in RNA-seq research."

Methodology lacks reference citation –

We have added information about methodology in abstract and add references for all the tools and methods used in the methodology section.

Table 1 and table 2 looks like a general information

• Without employing functional assays (such as qPCR, protein quantification, or immunological assays) to validate important differentially expressed genes (DEGs), the study only used transcriptome data (such as RNA-Seq).

We agree that functional assays, such as qPCR, protein quantification, or immunological assays, are crucial for further validating the differentially expressed genes (DEGs). However, the transcriptome data presented in Tables 1 and 2 provide an important foundation for identifying potential targets for further investigation. These data allow us to prioritize genes of interest and generate new hypotheses, which can then be tested using functional assays in subsequent studies. The transcriptome analysis serves as an essential starting point for understanding the molecular mechanisms involved and guiding future experimental approaches, which was the primary objective of this work.

• Despite the rat genome's comparatively good annotation, some genes or isoforms—especially those pertaining to immune-neural interactions—may still be missing functional annotations. This may restrict how DEGs are interpreted and how they might affect immune system function.

This limitation may indeed affect the interpretation of some DEGS and their potential role in the functioning of the immune system. However, we have made efforts to interpret the data obtained based on the most recent available annotations, and where data on the functions of genes are lacking, we have considered alternative approaches to hypothesize the functional significance of these genes.

• The study might find DEGs linked to immune function, but it doesn't investigate the fundamental processes that connect immune system control with amygdala excitability. The results might not give a good picture of how these DEGs affect immune-neural connections in the absence of mechanistic knowledge.

We agree that identifying DEGs related to immune function is only the first step towards understanding the relationship between molecular genetic and phenotypic differences. Nevertheless, the purpose of our study was to create a basis for further study of these relationships. We understand that our research does not cover the mechanisms, but the data obtained can serve as a basis for formulating hypotheses that will guide future experiments aimed at elucidating specific pathways and interactions. This will be an important topic for future research.
