# Supplementary material for: Аmygdala DEGs are associated with the immune system function: A comparative transcriptomic study of high- and low-excitability rat strains
Source: PLoS One. 2025 May 15;20(5):e0323325. doi: 10.1371/journal.pone.0323325 (PMC12080833; doi:10.1371/journal.pone.0323325)
Supplement: Attachment — Submitted filename: Response to Reviewer1.docx [file pone.0323325.s005.docx]

Reviewer #1:

Line 201 -- a random period mark is there, it should be removed.

Line 427-432: This is only 2 sentences, so I do not think it should be made as separate paragraphs.

Thank you for reading the manuscript carefully, we have corrected both technical errors in our text.
